# Synchronous parathyroid carcinoma and papillary thyroid carcinoma

**DOI:** 10.1002/ccr3.6369

**Published:** 2022-09-24

**Authors:** Ibtissem Ben Nacef, Dayssem Khelifi, Mehdi Kalthoum, Imen Rojbi, Ines Riahi, Sabrine Mekni, Mamia Ben Salah, Nadia Mchirgui, Karima Khiari

**Affiliations:** ^1^ Department of Endocrinology University Hospital of Charles Nicolle Tunis Tunis Tunisia; ^2^ Department of Otorhinolaryngology‐Head and Neck Surgery University Hospital of Charles Nicolle Tunis Tunisia

**Keywords:** hypercalcemia, hyperparathyroidism, nonmedullary thyroid carcinoma, papillary carcinoma, parathyroid carcinoma

## Abstract

The simultaneous occurrence of parathyroid carcinoma and nonmedullary thyroid carcinoma is unusual. We report the case of 60‐year‐old woman who was found to have concurrent parathyroid carcinoma with severe clinical manifestations of primary hyperparthyroidism in addition to an incidental papillary thyroid carcinoma. Parathyroid hormone level was 569 pg/ml (normal range 10–65), and the serum calcium concentration was 13.83 mg/dl (normal range, 8.8–10.4). Preoperative investigation found a large 3 cm anterior cervical nodule suggestive of parathyroid adenoma. Total thyroidectomy and left parathyroidectomy were performed, and the final anatomopathological examination of the operative specimen concluded the coexistence of papillary microcarcinoma and parathyroid carcinoma. Although parathyroid carcinoma is an uncommon cause of hypercalcemia, it should be considered when severe hypercalcemia is observed, and in case of coexistence of thyroid nodules. The possibility of both malignancies must also be considered since parathyroid and nonmedullary thyroid carcinoma rare cases have previously been reported.

## 1 INTRODUCTION

Parathyroid carcinoma is a rare endocrine malignancy, rarer when synchronous with a nonmedullary well‐differentiated thyroid carcinoma.^1^ Parathyroid carcinoma (PC) accounts of 0.005% of all malignant tumors, and it is responsible for less than 1% of primary hyperparathyroidism.^2^, ^3^

The initial manifestation of parathyroid carcinoma is usually quite acute, and the serum calcium and parathyroid hormone (PTH) levels are often higher than with parathyroid adenoma.^4^ Concurrent thyroid carcinoma in these patients is extremely rare, and to our knowledge, only a few 15 cases have been described previously in published reports.^1^, ^5^, ^6^ In 1974, the first case of concomitant thyroid and parathyroid disease was reported.^7^ The association between hyperparathyroidism and nonmedullary well‐differentiated thyroid carcinoma is found in 2.4%–3.7% of hyperparathyroidism cases.^6^, ^8^ However, coexistence of primary hyperparathyroidism due to parathyroid carcinoma with thyroid carcinoma is extremely rare. Here, we describe a patient with this uncommon presentation.

## 2 CASE REPORT

A 60‐year‐old woman with a history of diabetes mellitus type 2, hypertension, and no family history of thyroid cancer or multiple endocrine neoplasia type 1 or 2 presented with symptoms of severe hypercalcemia (confusion, acute renal failure, dehydration). Her calcium level was at 13.83 mg/dl (reference range, 8.8–10.4) with an increased serum parathyroid hormone level 569 pg/ml (reference range 10–65) (Table 1).

**TABLE 1:** Laboratory findings

Laboratory values	At admission	At 6 months postoperatively	Normal range
Calcemia mg/dl	13.83	9	8.8–10.4
Phosphatemia (mg/dl)	2.28	2.64	2.4–5
Parathyroid Hormone (pg/ml)	569	22	10–65
Albumin (g/L)	36	38	35–50
Magnesium(mg/dl)	2.2	2.1	1.9–2.7
Creatinine (μmol/L)	158	88	55–115
25‐OH‐Vitamin D (ng/ml)	22	31	20–40
Calcitonin (pg/ml)	1	‐	<5

Physical examination revealed a 3 cm basal cervical swelling lateralized to the left with absence of recurrent nerve palsy or cervical lymphadenopathy.

The cervical ultrasound showed a multinodular thyroid gland with 4–10 mm highly hypoechoic nodules classified as EU‐TIRADS five associated with a large nodule at the upper pole of the left thyroid which measured 3 cm evoking a parathyroid mass. Technetium‐99 m methoxyisobutylisonitrile (MIBI) cervical and mediastinal subtraction scintigraphy scans revealed increased uptake of left polar superior area of thyroid. A dual‐energy X‐ray absorptiometry test performed showed no evidence osteoporosis. Blood and urine tests and calcitonin levels were normal, making multiple endocrine neoplasia type 2 very unlikely.

**FIGURE 1:**
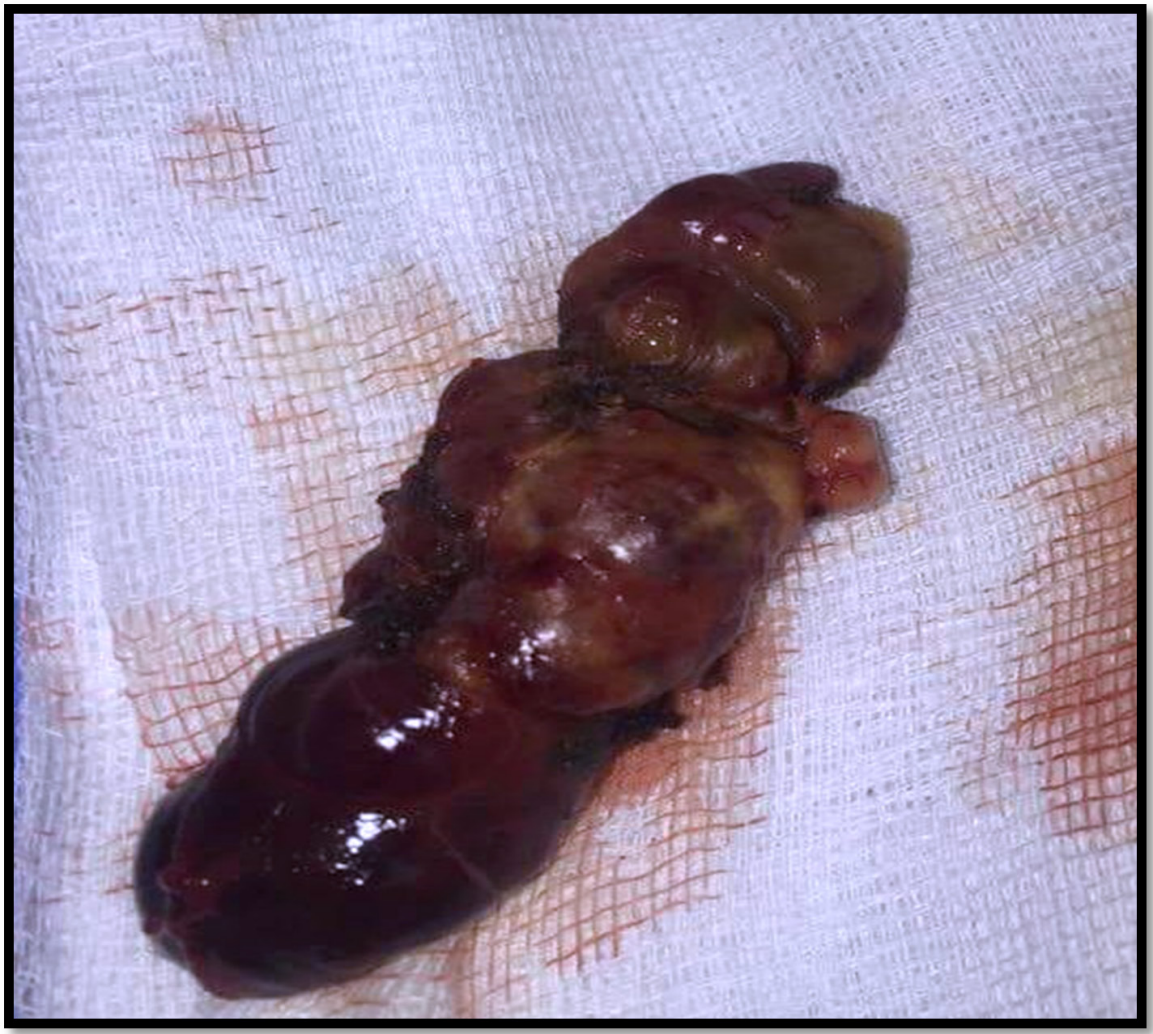
Surgical specimen. Surgical specimen from the patient (total thyroidectomy + left parathyroidectomy).

**FIGURE 2:**
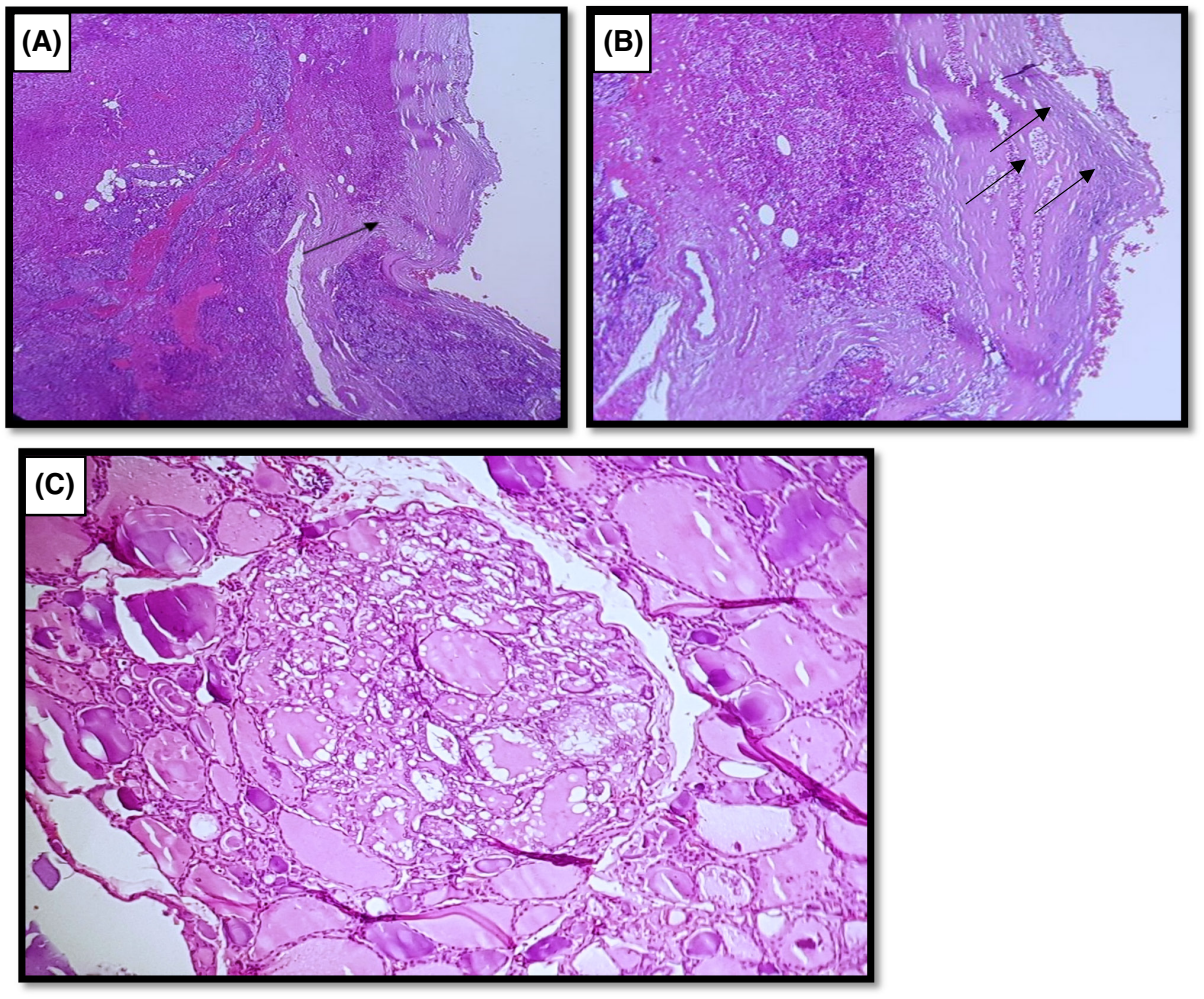
Histopathological finding. (A) Parathyroid tumoral nodule with thick capsule with incomplete capsular intrusion (arrow). The tumor cells had abundant cytoplasm with a slightly atypical rounded nucleus. Mitoses were estimated at 5 mitoses/10 CFG (hematoxylin–eosin stain original magnification 40×). (B) Nodular tumor proliferation (parathyroid). In places, there were images of capsular rupture and invasion of peri‐glandular adipose tissue with images of lymphatic‐vascular tumor emboli (arrows) (hematoxylin–eosin stain original magnification 40×). (C) Papillary microcarcinoma follicular variant. Histological evaluation demonstrating typical features of a papillary carcinoma with a follicular architectural pattern (hematoxylin–eosin stain original magnification 100×).

The patient received intravenously administered fluids and diuretics. Then, a total thyroidectomy, left parathyroidectomy, and central and lateral cervical lymph node dissection were performed in consideration of extemporaneous histological examination suggestive of a papillary microcarcinoma of the left lobe (Figure 1) and the suspicious aspect of the 4 cm parathyroid formation which was hard and adherent to the thyroid and difficult to dissect from the vascular axis (Figure 2A).

The definitive anatomopathological examination of the operative specimen showed a bifocal papillary microcarcinoma with vesicular component, with absence of central ganglion metastases. The left parathyroid gland was the site of significant multinodular hyperplasia with central nodule involvement with vascular emboli and capsular invasion (Figure 2B,C).

Considering the severity of the hypercalcemia and macroscopic appearance as well as the histological aspect, the diagnosis of parathyroid carcinoma was retained. Postoperatively, the patient was prescribed orally administered calcium and vitamin D3 and had normocalcemia in conjunction with PTH levels measured 30 pg/ml. Investigations showed no distant metastasis. Subsequently, the patient has remained normocalcemic, and the most recent serum calcium level (at 6‐month postoperative follow‐up) was 9 mg/dl (Table 1).

## 3 DISCUSSION

Although parathyroid carcinoma is not a usual cause of primary hyperparathyroidism,^1^, ^2^, ^9^ its incidence is increasing due to increased incidence of diagnosed primary hyperparathyroidism.^10^ Many presenting features of parathyroid hormone‐dependent hypercalcemia that should suggest the etiology of parathyroid carcinoma are shown in Table 2.^11^ The presence of some of these features in our patient, particularly the high calcium and PTH levels in serum, alert before surgery to the possibility of malignancy. After surgery, some anatomopathological features such as trabecular architecture, thick capsule, fibrous trabecular traversing the gland, nuclear atypia, and high mitotic rates should be looked for in parathyroid carcinoma, but none of these changes the diagnostic.^12^ The most definitive diagnostic criteria for parathyroid carcinoma are invasion of adjacent tissues capsular and vascular invasions (found in our case), involvement of regional lymph nodes, and distant metastatic lesions (not found in our case). The fibrotic pattern of the tumor invading the adjacent thyroid tissue in the setting of obvious capsular rupture in our patient makes the diagnosis of parathyroid carcinoma high likely. The size of the tumor, the infiltrative margin, and the trabecular pattern seen in this tumor are all features of parathyroid carcinoma.

**TABLE 2:** Manifestations suggesting parathyroid carcinoma in primary hyperparathyroidism

Symptomatic hypercalcemia
Hypercalcemia: Serum calcium >3.5 mmol/L
High serum PTH level: > 3–10 times above the upper limit of normal
Palpable neck mass
Simultaneous renal and overt skeletal involvement
Coexisting with recurrent severe pancreatitis and anemia
Recurrent laryngeal nerve palsy in patients without previous neck surgery

Synchronous parathyroid and nonmedullary thyroid carcinomas are extremely rare with only 15 previous cases reported in literature,^1^, ^5^, ^6^, ^7^, ^13^, ^14^, ^15^, ^16^, ^17^, ^18^, ^19^, ^20^, ^21^, ^22^, ^23^ and no correlation, up to date, has been discovered between these entities.^24^ Twelve patients (84%) were females with relatively young age (mean age of 56 years). Parathyroid carcinoma was active in 13 of the 15 patients described (including our patient): One published report described an occult parathyroid carcinoma that was found incidentally during total thyroidectomy.^15^ The lowest serum calcium level reported in the literature with parathyroid carcinoma has been 12.2 mg/dl, and the lowest reported PTH level associated with parathyroid carcinoma has been 159 pg/ml in conjunction with a serum calcium level of 13.3 mg/dl.^1^ Most of the reported cases with active parathyroid tumor had severe hypercalcemia and elevated PTH levels to up 100 times normal levels. The average size of the parathyroid carcinoma was 3.2 cm (maximum >5 cm) with two cases of concomitant hyperplasia or parathyroid adenoma. Synchronous thyroid carcinomas were papillary carcinoma type in 13 patients and follicular type in two cases.

Previous reports have suggested that a history of neck irradiation, long‐standing secondary HPT, end‐stage renal disease, hereditary HPT‐jaw tumor syndrome, and mutations of parafibromin (HRPT2) and cyclin D1 (CCND1) increase the risk of parathyroid carcinomas.^25^ Our patient has not been tested for the HRPT2 mutation, and she had no risk factors for parathyroid or thyroid carcinomas.

In case of severe hypercalcemia, parathyroid carcinoma should be considered as possible etiology, and if the peroperative frozen section diagnosis supports the suspicion of parathyroid carcinoma, an en‐bloc resection of the parathyroid tumor must be performed. For our patient, an en‐block resection was performed with central and lateral cervical lymph node dissection. Postoperatively, the patient has remained normocalcemic with normal PTH levels; however, she needs more follow‐up to assume that she does not have any remaining hyperfunctioning gland. In the previous described cases, the follow‐up of the patients ranged from 6 months to 6 years: most of the patients remained with normocalcemia, only one case presented persistent hypercalcemia, and another patient died from metastatic parathyroid carcinoma.^13^

## 4 CONCLUSION

In conclusion, coexistence of primary hyperparathyroidism due to parathyroid carcinoma along with nonmedullary thyroid carcinoma is extremely rare. Our present case report illustrates crucial points. In fact, although parathyroid carcinoma is an unusual cause of primary hyperparathyroidism, it should be given full attention. The diagnosis is generally established on the conjunction of clinical, radiological, biological, and histological signs.

Moreover, the fortuitous concomitant discovery of papillary thyroid carcinoma emphasizes the importance of knowing this association given the therapeutic implications that result from it.

## AUTHOR CONTRIBUTIONS

I.B.N, D.K, and M.K involved in data, information collection, manuscript preparation, manuscript review, and literature review. I.R, I.R, S.M, M.B.S, N.M, and K.K involved in study conception and contributed to manuscript draft.

## CONFLICT OF INTEREST

The authors declare no conflicts of interest.

## CONSENT

Written informed consent was obtained from the patient for the publication of this case report.

## Data Availability

No data were available.
